# Combining electro- and magnetoencephalography data using directional archetypal analysis

**DOI:** 10.3389/fnins.2022.911034

**Published:** 2022-07-29

**Authors:** Anders S. Olsen, Rasmus M. T. Høegh, Jesper L. Hinrich, Kristoffer H. Madsen, Morten Mørup

**Affiliations:** ^1^Department of Applied Mathematics and Computer Science, Technical University of Denmark, Lyngby, Denmark; ^2^WS Audiology, Lynge, Denmark; ^3^Danish Research Centre for Magnetic Resonance, Centre for Functional and Diagnostic Imaging and Research, Copenhagen University Hospital Amager and Hvidovre, Hvidovre, Denmark

**Keywords:** archetypal analysis, microstates, electroencephalography, magnetoencephalography, multimodal integration, spatiotemporal variability, directional statistics, Watson distribution

## Abstract

Metastable microstates in electro- and magnetoencephalographic (EEG and MEG) measurements are usually determined using modified *k*-means accounting for polarity invariant states. However, hard state assignment approaches assume that the brain traverses microstates in a discrete rather than continuous fashion. We present multimodal, multisubject directional archetypal analysis as a scale and polarity invariant extension to archetypal analysis using a loss function based on the Watson distribution. With this method, EEG/MEG microstates are modeled using subject- and modality-specific *archetypes* that are representative, distinct topographic maps between which the brain continuously traverses. Archetypes are specified as convex combinations of unit norm input data based on a shared generator matrix, thus assuming that the timing of neural responses to stimuli is consistent across subjects and modalities. The input data is reconstructed as convex combinations of archetypes using a subject- and modality-specific continuous archetypal mixing matrix. We showcase the model on synthetic data and an openly available face perception event-related potential data set with concurrently recorded EEG and MEG. In synthetic and unimodal experiments, we compare our model to conventional Euclidean multisubject archetypal analysis. We also contrast our model to a directional clustering model with discrete state assignments to highlight the advantages of modeling state trajectories rather than hard assignments. We find that our approach successfully models scale and polarity invariant data, such as microstates, accounting for intersubject and intermodal variability. The model is readily extendable to other modalities ensuring component correspondence while elucidating spatiotemporal signal variability.

## 1. Introduction

Brain function may be understood in terms of metastable states of activity involving anatomically distinct brain areas working in synchrony. Metastability refers to the brain lingering in a state before switching to another state. In functional magnetic resonance imaging (fMRI) literature, dynamic functional connectivity has revealed brain connectivity states using unsupervised machine learning methods (Cabral et al., 2017; Preti et al., 2017), and elucidated how the activity of these states varies following perturbations to the resting state, e.g., sleep (Stevner et al., 2019) or the administration of psychedelic drugs (Lord et al., 2019; Olsen et al., 2021). However, the frequency content in blood-oxygen-level-dependent (BOLD) fMRI is limited to very slow oscillations (<0.1 Hz) and thus does not allow for investigation of “real-time” brain state transitions and complicates, for instance, the analysis of evoked responses.

In electro- and magnetoencephalography (EEG and MEG), metastable states of sub-second activity span, denoted microstates, have been a research topic for many years (Lehmann, 1971; Lehmann et al., 1987)—see Khanna et al. (2015) and Michel and Koenig (2018) for reviews. Rather than involving specific brain regions, microstates are defined by whole-brain dipolar topographic maps. Microstates may be defined in a multitude of ways, including characterizations by principal and independent component analysis (Skrandies, 1989; Makeig et al., 1999), modified *k*-means (Pascual-Marqui et al., 1995), hidden Markov modeling of MEG power envelopes (Quinn et al., 2018; Coquelet et al., 2022) or agglomerative hierarchical clustering methods (Murray et al., 2008; Khanna et al., 2014). Of particular interest is the polarity invariance of the topographic maps; as M/EEG signals are naturally oscillating, the same microstate may be active although the sign of the input data changes (i.e., maxima become minima and vice versa) (Poulsen et al., 2018). In addition, the global scaling of the topographic maps is usually also irrelevant—it is, rather, the electrode activity relative to other electrodes that is important (Van De Ville et al., 2010). The current gold standard microstate analysis involves modified *k*-means clustering of instantaneous activity maps assessed using, e.g., global field power (Skrandies, 1990). Prototypes are constrained to unit norm, and the angle from data points to the prototypes is squared to account for polarity invariance. Other interesting models include Leading Eigenvector Dynamics Analysis (Cabral et al., 2017), which, although previously unused in EEG modeling, models interregional coherence by assessing the eigenvector of instantaneous coherence maps. Eigenvectors are axially symmetric unit vectors (scale-free) and may be modeled using diametrical clustering (Dhillon et al., 2003) to account for polarity invariance (Olsen et al., 2021).

The notion of meta-stability of EEG microstates has recently been challenged by Mishra et al. (2020) and Dekker et al. (2021) arguing that the brain traverses microstates in a continuous rather than discrete fashion. Thus, models that assign data to prototypes in an all-or-nothing fashion, such as *k*-means, may be too simple. As such, there is a need for methods that model data as traversing through continuous trajectories between states. A solution to this problem would be to define state topographies by extreme data points and describe brain activity as continuous navigation in the convex hull spanned by these states. Such a model has yet to be established for EEG and MEG microstates.

Another topic of interest in the analysis of brain function is multimodal integration. While EEG and MEG measure the same sources in the brain, i.e., synchronized postsynaptic currents in the dendrites of cortical pyramidal neurons, the electric potential and the normal component of the magnetic field of a tangential current source are rotated 90° relative to each other (Lopes da Silva, 2013). Furthermore, EEG and MEG complement each other regarding radially oriented sources, sensitivity to source depth, and tissue-specific signal attenuation. The combination of EEG and MEG is known to improve source localization accuracy (Sharon et al., 2007). Several approaches to M/EEG fusion have been suggested, including the use of Kalman filtering (Hamid et al., 2013), modeling modality dissimilarity correlations (Cichy et al., 2016), modality-specific error weighting using Bayesian optimization (Henson et al., 2009), and maximum entropy on the mean framework (Chowdhury et al., 2015). Although M/EEG integration is well-described in the literature, multimodal microstate analysis has not previously been explored.

Here we introduce directional archetypal analysis (DAA) and apply it for the joint integration of simultaneously recorded event-related potential (ERP) EEG and MEG data. Archetypal analysis (AA) is an unsupervised learning method for finding interpretable patterns in high-dimensional data. AA determines extreme points, denoted archetypes, that reside on the convex hull of the data cloud and determines how to express the data as convex combinations of such archetypes optimally. The determined archetypes can be considered distinct characteristics, forming prominent corners of the data (Cutler and Breiman, 1994). Thus, AA deviates from *k*-means that determine prototypical points or centers of the data cloud. Similarly, Hidden Markov Models, which model continuous transitions between states, also estimate prototypes rather than archetypes (Vidaurre et al., 2017). AA has been applied successfully in a variety of fields, including astronomy (Chan et al., 2003), survey and performance data (Seth and Eugster, 2016), chemistry and collaborative filtering (Mørup and Hansen, 2012), bio-informatics (Thøgersen et al., 2013; Hart et al., 2015), and neuroimaging (Mørup and Hansen, 2012; Hinrich et al., 2016; Cona et al., 2019; Krohne et al., 2019), including for the analysis of single-trial electroencephalography (EEG) brain response variability (Tsanousa et al., 2015).

While conventional AA determines archetypes based on a least-squares loss function of the reconstruction, we here reformulate the method to account for axially symmetric spherical data using a distance measure derived from the Watson distribution (Watson, 1965; Sra and Karp, 2013). By projecting every measured data point onto a (*D*−1)-dimensional sphere (*D* being the number of electrodes or magnetometers), we ensure that the decomposition is not driven by the scale of the input data. Similarly, by employing a statistical distribution that models diametrically opposite points as equal, we also directly model the polarity invariance of the input data. We demonstrate the utility of the developed method for the joint modeling of EEG and MEG ERPs, ensuring component correspondence while accounting for the shared modality-wide complementary information regarding how the extracted sources are spatiotemporally elicited in the two modalities. We use a similar approach to Hinrich et al. (2016) for the modeling of multisubject data utilizing a shared archetype-generating mechanism across subjects while allowing for subject-specific archetypes and mixing matrices. Specifically, we conduct multimodal integration by identifying shared archetypal temporal profiles used to generate the archetypes while determining the modality- and subject-specific expression of these shared temporal profiles.

In summary, we propose the DAA model accounting for scale- and sign-invariant modeling of EEG and MEG data as well as their joint integration, assuming the timing of the neural responses to stimuli are consistent across EEG and MEG. Based on the developed DAA we demonstrate:

(i) The merits of DAA as opposed to conventional AA when data resides on the unit (hyper-)sphere.
(ii) The merits of DAA as opposed to a DAA-clustering model with hard assignments.
(iii) How DAA can be used to model microstates in evoked response EEG and MEG data.
(iv) How DAA can be used for the joint integration of EEG and MEG data.

The novelty of this work lies both in the development of a new AA framework for directional statistics (DAA) as well as a novel approach for multimodal integration of EEG and MEG accounting for spatiotemporal variability while ensuring component correspondence across modalities as defined by an assumed shared timing of the responses to stimuli.

## 2. Methods

### 2.1. Data

Analysis was carried out on the openly available multimodal face perception data set introduced by Wakeman and Henson (2015) with concurrent EEG and MEG recordings in 19 subjects (8 females), whom all provided written informed consent. The study was originally approved by the Cambridge University Psychological Ethics Committee, and the data is openly accessible through OpenNeuro (accession number: ds000117, version 1.0.4^1^). Each participant completed six sessions where they were presented with approximately 150 images of famous, unfamiliar, or scrambled (head shape preserved but face unrecognizable) faces. Each functional trial started with the appearance of a fixation cross for a random duration (400–600 ms) and then a stimulus (face or scrambled face, 800–1,000 ms). Between stimuli, a white circle was shown for 1,700 ms. Across the experiment, participants were told to focus on a fixation cross at the center of the screen and refrain from blinking during stimulus presentation. All faces were shown twice, either immediately after or following 5–15 other stimuli (50/50 of each).

MEG and EEG data were recorded simultaneously using an Elekta Neuromag Vectorview 306 system (Helsinki, FI) with 102 magnetometers and a 70-channel Easycap EEG cap with the reference electrode on the nose. The common ground electrode was placed at the left collar bone. Electrooculograms, both vertical and horizontal, were measured using two sets of bipolar electrodes, and similarly for electrocardiogram with electrodes at the left lower rib and right collarbone.

### 2.2. Preprocessing

Data from 16 subjects (the data set authors excluded three subjects due to poor data quality) were provided in a maxfiltered version and were subsequently preprocessed in Fieldtrip (Oostenveld et al., 2011) using modified processing scripts provided by Robert Oostenveld^2^. Our pipeline consisted of (1) epoching the data according to trials and conditions, (2) rejecting epochs with EEG, MEG, or electrooculography artifacts, (3) bandpass filtering the data between 0.5 and 40 Hz, (4) modality-wise principal component analysis retaining the first 50 components and subsequently subtracting the channel-wise mean, and (5) downsampling the data from 1, 100 Hz to 200 Hz. Finally, trials were averaged within-subject over multiple presentations of the three stimuli: familiar, unfamiliar, and scrambled.

### 2.3. Archetypal analysis

In the classic archetypal analysis, we seek a decomposition **X**≈**AS** of a data matrix **X**∈ℝ^*D*×*N*^, where *N*∈ℕ corresponds to the number of observations and *D*∈ℕ corresponds to the dimensionality (e.g., number of channels) (Cutler and Breiman, 1994). The decomposition determines a set of archetypes $A=\tilde{\text{X}}\text{C}$, which are weighted combinations of the matrix $\tilde{\text{X}}$ that, as introduced in Hinrich et al. (2016), may differ from the input matrix **X**, e.g., through some transformation, and a mixing matrix **S**. The two matrices **C**∈ℝ^*N*×*K*^ and **S**∈ℝ^*K*×*N*^ (where *K*∈ℕ corresponds to the number of archetypes to be extracted) are used to reconstruct the data matrix, and we denote the reconstruction $\hat{\text{X}}=\tilde{\text{X}}\text{CS}\in {\mathbb{R}}^{D\times N}$. In this formulation, the archetypes are found by convex combination (weights sum to one) of the existing data points in $\tilde{\text{X}}$ by matrix multiplication with **C**, such that the archetypes are defined by the columns of the matrix $A=\tilde{\text{X}}\text{C}$. Each observation in the reconstruction $\hat{\text{X}}$ is then defined in terms of a convex combination of these archetypes given by the columns of **S**.

For some measure of distance between the data and reconstructions, *D*(°|°), the problem of identifying **C** and **S** can be formulated as:


(1) $$\begin{array}{l} \text{argmin}\left(D(\text{X}|\hat{\text{X}})\right)\\ \text{ C},\text{S}\\ \;s.t.\;|c_{\cdot ,k}{|}_{1}=1,\;|s_{\cdot ,n}{|}_{1}=1,\\ \text{ C}\geq 0,\;\text{S}\geq 0, \end{array}$$

where **c**_·, *k*_ corresponds to column *k* in **C** (the *k*'th archetype generator), **s**_·, *n*_ corresponds to column *n* in **S** (the *n*'th observation), |·|_1_ is the ℓ_1_-norm which is constrained to one (i.e., sum of absolute values constrained to 1), and **C**, **S**≥0 enforces non-negativity in the elements of **C** and **S**. Together, the constraints ensure the archetypes and reconstructions are related through convex combinations (non-negative and sum to one). The problem is solved by alternately updating **C** and **S** (i.e., alternately finding optimal archetypes for a given expression **S** of the archetypes, and finding optimal expression of the archetypes given the definition of archetypes by **C**). The classic Euclidean distance measure amounts to a least squares loss, *ℒ*_*ls*_, and can be expressed using the Frobenius norm as: $D(\text{X}|\hat{\text{X}})=||\text{X}-\tilde{\text{X}}\text{CS}|{|}_{F}^{2}.$.

Whereas the Euclidean AA implicitly assumes normally distributed noise, the AA has been advanced to other types of data sets, including binary (Bernoulli likelihood) and integer variables (Poisson likelihood) (Seth and Eugster, 2016) as well as ordinal responses (Fernández et al., 2021). However, no generalization of AA in the context of directional statistics currently exists.

### 2.4. Directional archetypal analysis

In the current treatment of directional archetypal analysis (DAA), we focus on axially symmetric data as characterized by the Watson distribution with the probability density function:


(2) $$W(x|\mu ,\kappa )=c_{D}(\kappa )\exp(\kappa {\left(\mu^{T}x\right)}^{2}),$$

where **x**∈*S*^*D*−1^ (the (*D*−1)-dimensional unit hypersphere), **μ** defines a mean direction, κ defines a concentration around that mean direction, and *c*_*D*_(κ) is a normalization constant (see Watson, 1965). Specifically, we consider data where a direction, **x**, and its negative are equivalent (invariance to sign flip), which corresponds to **x**∈ℙ^*D*−1^, where ℙ^*D*−1^ is the (*D*−1)-projective hyperplane (Sra and Karp, 2013).

Instead of a Euclidean distance (least squares) loss, the Watson distribution measures the squared difference in the angle between the reconstruction and the corresponding data point. Contrary to classic archetypal analysis, we will investigate angular properties between observations that lie on the surface of the unit hypersphere, i.e., if the *n*'th observation in the data matrix **X** is denoted **x**_*n*_, then we can reparameterize any observation as ${\text{x}}_{n}=\sqrt{\kappa_{n}}{\tilde{\text{x}}}_{n}$ such that ${\tilde{x}}_{n}\in S^{D-1}$ with precision $\kappa_{n}=||x_{n}|{|}_{2}^{2}$. Notably, the precision κ_*n*_ can thereby be absorbed in **x**_*n*_ by scaling ${\tilde{\text{x}}}_{n}$ by $\sqrt{\kappa_{n}}$. Thereby κ_*n*_ can be interpreted as the amount of precision assigned to the spherically distributed observations according to the Watson distribution given in (2). By optimizing with respect to the original data **x**_*n*_ (1), emphasis will be given to the reconstruction ${\tilde{\text{x}}}_{n}$ with high precision κ_*n*_ while ensuring that the archetypes themselves are not influenced by scale-difference in data. We further assume that diametrically opposed ${\tilde{\text{x}}}_{n}$ are equivalent, and thus that ${\tilde{x}}_{n}\in {\mathbb{P}}^{D-1}$. For each observation, the angle (in *D*-dimensional space) can be measured as the inner product of the reconstruction (normalized to have unit *l*_2_-norm) and the data points. We define the (unnormalized) reconstruction of **x**_*n*_ according to the AA model as ${\hat{x}}_{n}=\tilde{X}Cs_{n}$. The loss *ℒ*_*W*_, over *N* points is then:


(3) $${ℒ}_{W}=-\sum_{n=1}^{N}{\left(\;{\text{x}}_{n}^{⊤}{\hat{\text{x}}}_{n}/||{\hat{\text{x}}}_{n}|{|}_{2}\right)}^{2}$$

Note that this loss function, while inspired by the Watson distribution, is not a density, and we do not, e.g., determine the normalization constant. To derive update rules for the DAA algorithm, we seek the derivative of the loss with respect to the model parameters **S** and **C**. We define two vectors of inner products **z** and **q** with elements $z_{n}=x_{n}^{⊤}{\hat{x}}_{n}$ and $q_{n}={\hat{x}}_{n}^{⊤}{\hat{x}}_{n}$ and denote the matrices with the elements of **z** and **q** in their diagonal as **D***z* = diag(**z**) and **D***q* = diag(**q**), respectively. Summing over all the squared angles between data and reconstruction can be written as the following loss (defining **V** = **D***z***D***q*^−1/2^):


(4) $${ℒ}_{W}=\text{ V}:\text{V},$$

where the colon operator “°:°” designates the inner product such that for matrices **A** and **B** we have that **A**:**B** = Tr(**A**^⊤^**B**). We will approach determining the scalar by matrix derivatives by initially working in the (total) differential form and then converting to canonical form^3^. Thus, to obtain the gradient of a scalar *ℱ*(**A**) w.r.t a matrix **A**, i.e., ∇_**A**_*ℱ*(**A**), we need to determine a matrix **B** such that δ*ℱ*(**A**) = Tr(**B**^⊤^δ**A**) = **B**:δ**A**, because then ∇_**A**_*ℱ*(**A**) = **B**. The differential of *ℒ*_*W*_ is then:


(5) $$\begin{array}{l} \delta {ℒ}_{W}=2\text{V}:\delta \text{V}=2\text{V}:\delta \left(Dz{\text{Dq}}^{-1/2}\right)\\ \;=2\text{V}:\delta {\text{DzDq}}^{-1/2}+2\text{V}:\text{Dz}\delta {\text{Dq}}^{-1/2} \end{array}$$

The gradients of *ℒ*_*W*_ w.r.t. **S** and **C** can then be found to be:


(6) $$\begin{array}{l} \delta {ℒ}_{W}(\text{C})=2\text{TV}:\tilde{\text{X}}\text{C}\delta S=2{\text{C}}^{⊤}{\tilde{\text{X}}}^{⊤}\text{TV}:\delta \text{S}\\ \;\Rightarrow \nabla_{\text{S}}{ℒ}_{W}=2{\text{C}}^{⊤}{\tilde{\text{X}}}^{⊤}\text{TV} \end{array}$$

(7) $$\begin{array}{l} \delta {ℒ}_{W}(\text{S})=2\text{TV}:\tilde{\text{X}}\delta \text{CS}=2{\tilde{\text{X}}}^{⊤}{\text{TVS}}^{⊤}:\delta \text{C}\\ \;\Rightarrow \nabla_{\text{C}}{ℒ}_{W}=2{\tilde{\text{X}}}^{⊤}{\text{T VS}}^{⊤}, \end{array}$$

where we defined $\text{T}={\text{X Dq}}^{-1/2}-\hat{\text{X}}{\text{ Dq}}^{-3/2}\text{ Dz}$.

For this application, we constrain $\tilde{X}$ to the hypersphere, i.e., we normalize every time-point for each modality, subject, and condition across channels. We introduce an additional constraint on $\tilde{X}\text{C}$ to ensure that the archetypes lie on the same hyper-hemisphere. We can ensure this by only allowing the archetypes to be constructed using a flipped version, ${\tilde{X}}_{f}$ of $\tilde{X}$ which is projected onto a chosen hyper-hemisphere. We determine the dominant hyper-hemisphere in the data by the first principal component. We then negate (“flip”) each data point if its projection onto this dominant direction is negative and obtain the archetypes as ${\tilde{X}}_{f}$. We also scale the data matrix **X** by its Frobenius norm (across all data points) for each subject and modality to ensure each subject and modality has a similar influence on the loss when considering the multisubject and multimodal modeling described next.

### 2.5. Multimodal multisubject directional archetypal analysis

Similar to how Hinrich et al. (2016) extended archetypal analysis to multisubject data, we extend DAA to parameterize multisubject and multimodal data sets. For modalities *m* = 1, …, *M* and subjects *b* = 1, …, *B*, we approximate our observed data matrices **X**^(*m, b*)^ as $X^{\left(m,b\right)}asX^{\left(m,b\right)}\approx {\tilde{X}}^{\left(m,b\right)}CS^{\left(m,b\right)}$. As such, our model contains a global archetype generator matrix **C** and modality- and subject-specific mixing matrices **S**^(*m, b*)^ as well as archetypes ${\tilde{X}}^{\left(m,b\right)}C$, while the archetypes are generated from the same convex combination of features. The loss function in (3) is thereby extended to multiple subjects and modalities by:


(8) $${ℒ}_{MW}=-\sum_{m=1}^{M}\sum_{b=1}^{B}\sum_{n=1}^{N}{\left({\text{ x}}_{n}^{(m,b)⊤}{\hat{\text{x}}}_{n}^{\left(m,b\right)}/||{\hat{\text{x}}}_{n}^{\left(m,b\right)}|{|}_{2}\right)}^{2},$$

where ${\hat{x}}_{n}^{\left(m,b\right)}={\tilde{X}}^{\left(m,b\right)}Cs_{n}^{\left(m,b\right)}$ and Equations (6) and (7) revised accordingly. We ensure the unit-norm of the columns of **C** and **S** by recasting the problem in *l*_1_-normalization invariant variables, as introduced in Mørup and Hansen (2012). For instance, for an element in **S**^(*m, b*)^, $s_{k,n}^{\left(m,b\right)}$, the recast parameter is ${\tilde{s}}_{k,n}^{\left(m,b\right)}=s_{k,n}^{\left(m,b\right)}/\underset{k^{\prime }}{\sum }s_{k^{\prime },n}^{\left(m,b\right)}$. We will omit the tilde for simplicity. We ensure non-negativity using a projected gradient method, which simplified amounts to a parameter update based on some step size μ and some gradient w.r.t. the distance defined above, $g_{k,n}^{\left(m,b\right)}$, as: $s_{k,n}^{\left(m,b\right)}\leftarrow \text{max}\left(s_{k,n}^{\left(m,b\right)}-\mu g_{k,n}^{\left(m,b\right)},0\right)$. For details regarding the projected gradient procedure, we refer to Mørup and Hansen (2012, Section 2.3).

In practice, we determine the gradient for **C** for every subject and modality and subsequently sum the gradients across these. We then update **C** and the step size μ_*c*_. That is, we decrease the step size with a factor $\frac{1}{2}$ if the new summed loss is worse than the previous one. If the new loss is improved, we slightly increase the step size (by a factor 1.1) and end the update. For **S**^(*m, b*)^, the gradient is once again determined for every subject and modality, though this time without summation across these. The loss for every time point, modality, and subject is computed, and corresponding elements of **S**^(*m, b*)^ are only updated if the new loss is lower than the previous one. Likewise, step sizes, which are specific to sample, subject, and modality, are increased/decreased (by the same factors as above) if the new loss is improved/worsened compared to the previous one.

In our implementation, we compute, for every update of the archetype generator matrix **C**, the matrices ${\left({\tilde{X}}^{\left(m,b\right)}C\right)}^{⊤}{\tilde{X}}^{\left(m,b\right)}C$ and $X^{(m,b)⊤}{\tilde{X}}^{\left(m,b\right)}C$ for fast computation of $D_{z}^{\left(m,b\right)}$ and ${\text{S}}^{\left(m,b\right)}$. This reduces the overall time complexity updating **S**^(*m, b*)^ substantially to be O(MBNK), whereas the overall time-complexity updating **C** is O(MBNK).

### 2.6. Multimodal multisubject directional clustering

In order to contrast the performance of the developed DAA to conventional clustering based on directional statistics as used in the modified *k*-means procedure of Pascual-Marqui et al. (1995) we further develop a hard clustering multimodal, multisubject clustering procedure inspired by the DAA. In conventional clustering, either modalities and subjects need to be modeled separately, or data merged, to ensure consistent centroids across subjects. By defining the cluster centroids in terms of a latent generator as in the DAA, it is possible to define a multimodal, multisubject hard assigned clustering procedure by endowing the DAA model with hard assigned clusters, i.e., by replacing the AA model formulation in (1) with ℓ_0_ constraints on **S** as opposed to ℓ_1_ constraints. Thereby the optimization of **S** changes to a *k*-means type assignment of observation to centroids according to the maximally squared inner product, i.e.,


(9) $$k^{*}={\text{argmin}}_{k}\left[-{\left({\text{ x}}_{n}^{(m,b)⊤}{\hat{\text{X}}}^{\left(m,b\right)}{\text{c}}_{k}/||{\hat{\text{X}}}^{\left(m,b\right)}{\text{c}}_{k}|{|}_{2}\right)}^{2}\right]$$

such that $s_{k,n}^{\left(m,b\right)}=1$ for *k* = k^*^ and 0 otherwise.

### 2.7. Model comparison and consistency

We evaluated DAA and our clustering approach using the Watson loss and conventional AA solutions across runs with sum of squared errors (SSE). While the Watson loss is given in (8), we assessed the least squares reconstruction error of the Euclidean AA model as


(10) $$\text{SSE}=\sum_{m}\sum_{b}||{\text{X}}^{\left(m,b\right)}-{\tilde{\text{X}}}^{\left(m,b\right)}{\text{CS}}^{\left(m,b\right)}|{|}_{F}^{2}.$$

To evaluate the consistency of the archetypal mixing, we employed normalized mutual information (NMI) similarly to Hinrich et al. (2016), since each column of **S**^(*m, b*)^ may be considered a probability distribution over components. For *k* = 1, …, *K* archetypes and two runs *r* and *r*′, NMI is here given by:


(11) $$\text{NMI}({\text{S}}^{r},{\text{S}}^{r^{\prime }})=\frac{2\text{MI}({\text{S}}^{r},{\text{S}}^{r^{\prime }})}{\text{MI}({\text{S}}^{r},{\text{S}}^{r})+\text{MI}({\text{S}}^{r^{\prime }},{\text{S}}^{r^{\prime }})}$$

(12) $$\text{MI}({\text{S}}^{r},{\text{S}}^{r^{\prime }})\;=\sum_{k,k^{\prime }}p(k,k^{\prime })\log\frac{p(k,k^{\prime })}{p(k)p(k^{\prime })}$$

(13) $$\begin{array}{rc} p\left(k,k^{\prime }\right) & =\frac{1}{N}\underset{k}{\sum }s_{kn}^{r}s_{k^{\prime }n}^{r^{\prime }}. \end{array}$$

NMI gives a score between 0 and 1 and is invariant to permutations of components. Here we compared losses and NMI between 5 runs of each model, where each model was compared to the preceding model. That is, comparisons were made between models 1−2, 2−3, …, 5−1 to avoid correcting for dependent comparisons if evaluating all model combinations. Presented NMI values are averages across subjects, modalities, and conditions. To minimize the effect of local minima, each run is the best of 100 randomly initialized models, where both **C** and **S** were initialized as rate 1 exponential random variables exp(1) normalized to the simplex.

## 3. Results

### 3.1. Three-dimensional illustration

**Figure 1:**
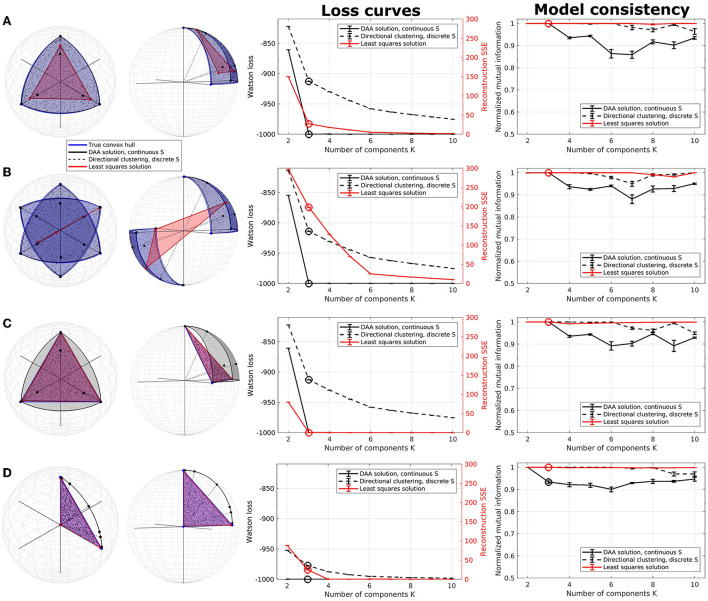
Illustration of directional archetypal analysis (DAA) vs. its Euclidean counterpart and a directional clustering model in three dimensions, including loss curves and normalized mutual information (NMI), which are evaluated on 5 models each being the best of 100 simulations. **(A)** Spherical data simulated using three true archetypes situating on the axis corners. The solutions obtained using conventional Euclidean archetypal analysis and directional archetypal analysis with *K* = 3 components and the convex hull spanned by the archetypes, as well as centroids determined using directional clustering, are also shown. **(B)** Spherical data simulated with three true archetypes situating on the axis corners as well as their diametrical opposite, i.e., polarity-invariant archetypes. **(C)** Non-spherical data simulated using the same three true archetypes as in **(A)**. **(D)** Data simulated with three true archetypes, of which one is the origin. Error bars represent standard error of the mean. Note that the loss functions for the models (Watson loss and sum of squared errors, respectively) are not directly comparable but are shown on the same graph to highlight corners in the loss indicating a potential optimal model.

To illustrate DAA, we applied it to four synthetic three-dimensional data sets, two of which were defined on *S*^2^, and contrasted the results obtained to the classic Euclidean AA approach and DAA modified to hard assignment, hereafter denoted directional clustering (see Figure 1). All three models were run in five sets of 100 random initializations of the matrices **C** and **S**, where the best model, in terms of loss, for each of the five runs was selected. In total, this leads to 500 model fits for each model and each number of estimated archetypes *K*.

The first synthetic dataset occupies one octant of the unit sphere with three natural corners constituting the archetypes. While DAA determines archetypes very close to the true archetypes and produces a convex hull on the surface of the sphere octant, the Euclidean solution produces archetypes further from the truth and a simplex-shaped principal convex hull encompassing the interior of the sphere (see Figure 1A). Directional clustering is even less flexible and locates centroids further away from the true archetypes. Due to the binary representation of the assignment matrix **S**, this solution corresponds to clustering, i.e., defining prototypes as opposed to archetypes. The loss curves indicate a deflection at *K* = 3 components (highlighted) for all three models. Whereas DAA converges to the true solution at *K* = 3, Euclidean AA and the directional clustering model show a less trivial loss curve gradually improving by including more components (i.e., clusters). The same models also have very high consistency for all component numbers. When *K*>3, extra DAA components become ambiguous and thus, model consistency decreases for this model, indicating that high model consistency is not necessarily equivalent to a well-performing model reconstruction.

In the second example, data were generated occupying two opposing octants of the unit sphere using the same true archetypes and their diametrical opposites, reflecting polarity invariant data (Figure 1B). The three models visualized using three components show vastly different results—while DAA remains able to produce a spherical principal convex hull close to the original solution defining a polarity invariant spherical convex hull, Euclidean AA is not able to produce a principal convex hull that encapsulates the data, identifying two archetypes along one direction and one archetype in the opposite hemisphere. Upon inspecting the loss curves, the Euclidean AA deflects at *K* = 6 components, i.e., double the number of true archetypes, whereas DAA and directional clustering bend at the expected *K* = 3 components. As such, Euclidean AA requires more components to explain polarity-invariant data. Similar to the former example, directional clustering with hard-assignment of states provides polarity-invariant centroids rather than data extremes defined by clusters at the interior of the spherical convex hull. Thus, for polarity invariant spherical data, DAA successfully provides a solution that determines archetypes defining a spherical convex hull that, through their convex combinations defined by the matrix **S**, optimally span the synthetic data points.

The third data set is simulated on the simplex spanned by the same three archetypes as in the first example, although without normalizing data to the sphere (Figure 1C). Both DAA, which projects data to the sphere before modeling, and Euclidean AA determine archetypes close to the true solution. Their loss curves also show similar deflection at *K* = 3, showcasing that when the data points are simulated on a non-spherical simplex, the two solutions produce similar archetypes although the simplex spanned by the DAA archetypes is spherical.

DAA and directional clustering project data points to the sphere surface prior to modeling, which may be problematic if the archetypes are far from the sphere surface. Especially if, in the extreme case, one of the archetypes is the origin. This case is exemplified in Figure 1D, where neither DAA nor directional clustering is able to extract sensible archetypes. To summarize, DAA and directional clustering may be used to model scale- and polarity-invariant data but suffer if the underlying convex hull is spanned by the origin.

### 3.2. Examination of event-related potential subject variability

**Figure 2:**
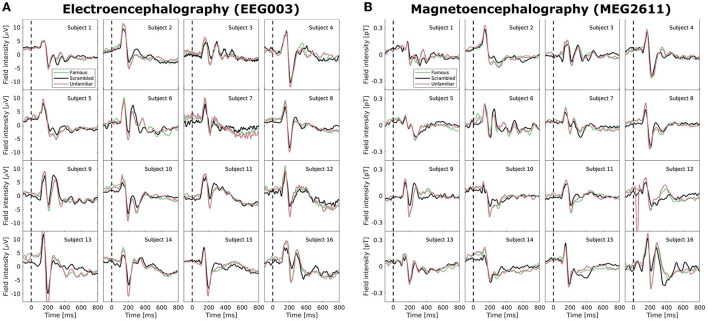
Average mean-subtracted time-locked subject event related potentials (ERPs) after preprocessing but without normalization for the three face perception conditions (familiar, unfamiliar, scrambled). **(A)** A selected electroencephalographic electrode (EEG003) located near the right occipital lobe. **(B)** A selected magnetoencephalographic magnetometer (MEG2611) located near the right temporal lobe.

Neuroimaging data, including EEG and MEG recordings of ERPs, carry large intra- and inter-subject variability. In Figure 2, we examined the Wakeman and Henson (2015) data set after pre-processing but before normalization; specifically, we highlight the EEG electrode with the largest amplitude (EEG003, located to the right of the occipital pole) and similarly for MEG (MEG2611, located near the right temporal lobe). The ERPs, which are averages of many time-locked trials within-subject, deviate vastly between subjects. While a positive deflection at approximately 100 ms and a stronger negative component at approximately 170 ms are generally visible for all subjects (as also reported in Wakeman and Henson, 2015), both scale and morphology of the ERP tend to vary. High variability is also visible in the post-170 ms positive deflections, and, for example, subjects 3, 6, 9, and 16 show sufficiently high positive deflections that they may even be considered a third ERP component. We observe very little consistent deviation between the three conditions (familiar, scrambled, unfamiliar). With the added difficulty of combining two modalities that display highly different topographies, a model that can account for inter-subject and inter-modal variability in microstate analyses is needed.

### 3.3. ERP data, unimodal

**Figure 3:**
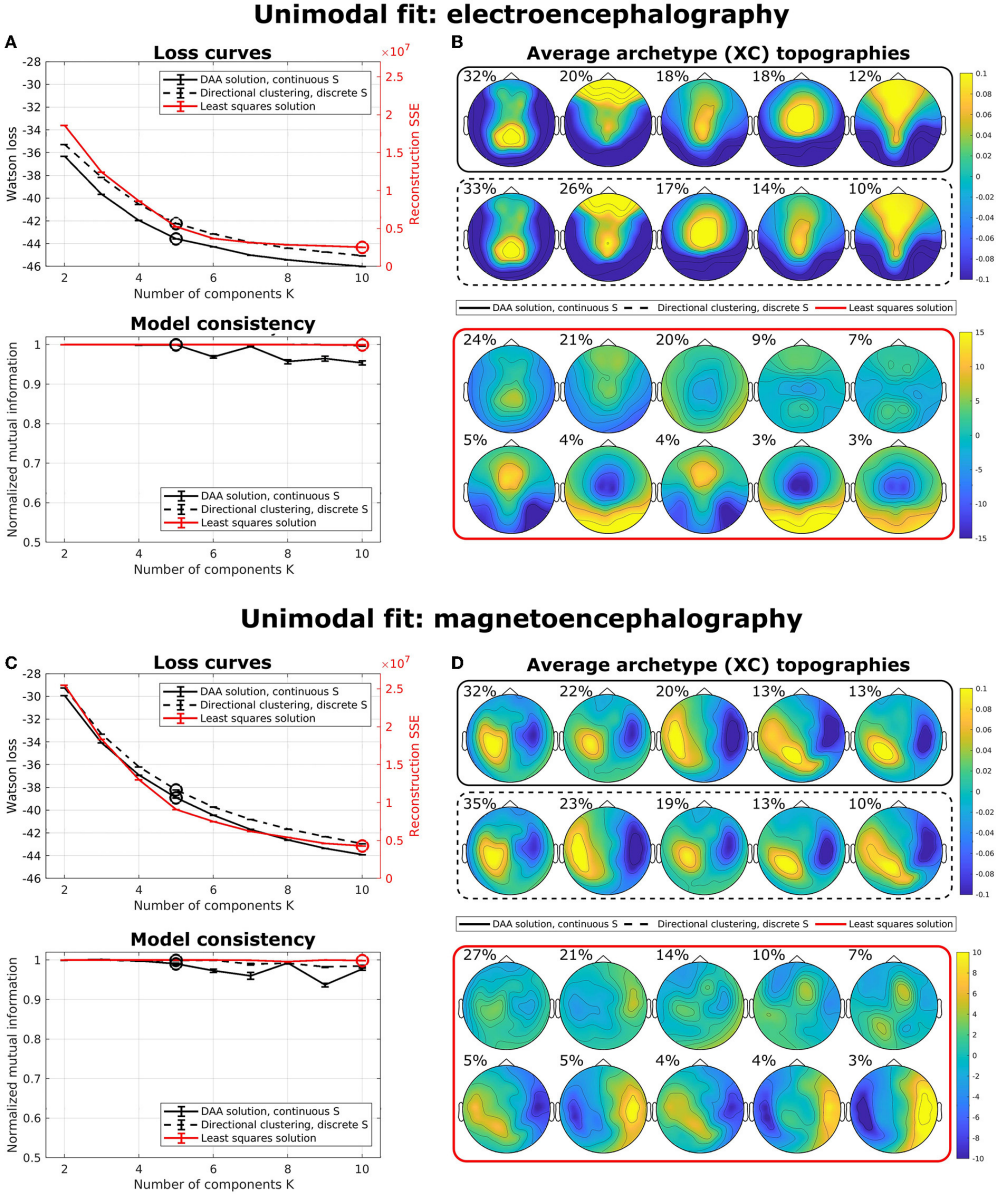
Unimodal multisubject archetypal analysis fits using DAA, conventional Euclidean archetypal analysis, and a directional clustering model derived from DAA using hard class assignment. **(A,C)** Loss curves and model consistency evaluated using NMI evaluated on 5 models, where each is the best of 100 model fits. Error bars represent standard error of the mean. **(B,D)** Archetype topographical maps for the best model fits with *K* = 5 and *K* = 10 for the directional models and the Euclidean model for EEG and MEG data, respectively. Archetypes are averaged across subjects and are ordered according to the total occupation of the archetypal mixing matrix **S**.

To illustrate the effects of multimodal fusion, we first applied our algorithms to unimodal data (i.e., data coming from a single modality) with a multisubject model. That is, we produced separate models for only EEG data and only MEG data. We compared our results to the multisubject AA model by Hinrich et al. (2016) with a least-squares loss function. To minimize the effect of local minima, we ran our models 100 times with randomly (exponentially) sampled **C** and **S** and selected the model with the lowest loss. Figure 3 shows average loss curves and NMI for five such runs, with error bars representing standard error of the mean. The results show that, for both EEG (Figure 3A) and MEG (Figure 3C), the loss curves for all three models decrease steadily with an increasing number of components. DAA consistently shows improved loss compared to directional clustering with discrete state assignment. Figures 3B,D highlights the topographical maps for the determined archetypes for the models with the lowest loss for *K* = 5 and *K* = 10 for the directional and Euclidean models, respectively. The archetypes, which are averages across subjects and conditions, are ordered according to their percentage total occupation of the averaged archetypal mixing matrix **S**. Given the shared use of sign- and scale-invariance, the archetypes for DAA and the clustering equivalent are similar, with only a minor change in archetype proportion and ordering. The same results for Euclidean AA show some archetype duplications (e.g., archetypes 6 and 8). The AA archetypes vary more in scale, since this model explicitly models the scale of the data.

For both unimodal models, the NMI for the Euclidean implementation is very high, which indicates that this model is very stable upon selecting the best of 100 models to avoid local minima. However, model consistency is generally high for all three models.

### 3.4. ERP data, multimodal

**Figure 4:**
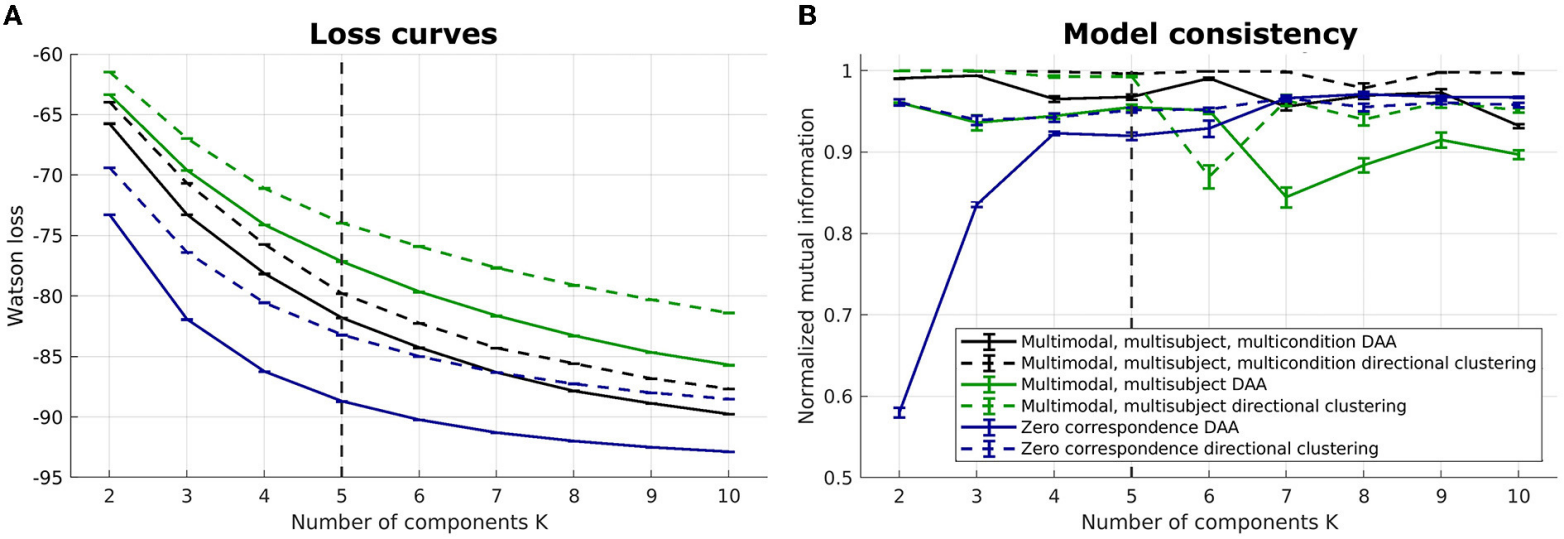
Loss curves and NMI for multimodal, multisubject DAA and directional clustering models. **(A)** loss curves for the six models, including multimodal, multisubject, multicondition DAA, a model where the three conditions are concatenated in time to enforce equal archetypes, and a model modeling all subjects separately with no correspondence between subjects. Error bars represent standard error of the mean. **(B)** NMI to inform on model consistency. Both loss curves and NMI are evaluated on 5 model fits for each model, each being the best of 100 fits.

**Figure 5:**
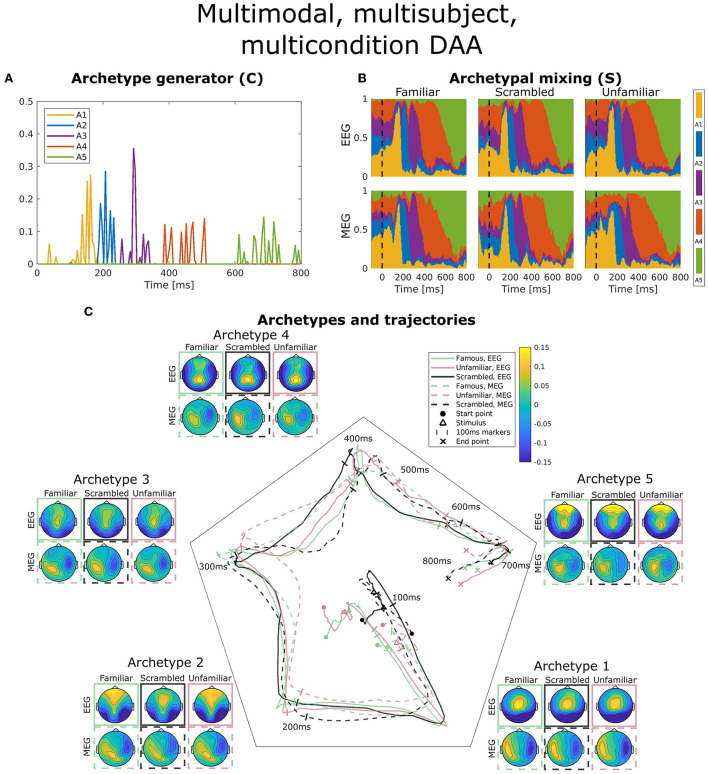
Visualization of the best obtained fit for multimodal, multisubject, multicondition DAA with *K* = 5 components. **(A)** The subject-, modality-, and condition-shared archetype generator matrix **C** with information on the specific samples from which archetypes are generated. **(B)** The archetypal mixing matrix **S** averaged across subjects showing how samples are probabilistically allocated to archetypes. The mixing matrix has been smoothed with a rectangular window of size 3 samples. **(C)** Archetype trajectory averaged across subjects based on the mixing matrix **S** smoothed with a rectangular window of 10 samples as well as average archetype topographical maps.

**Figure 6:**
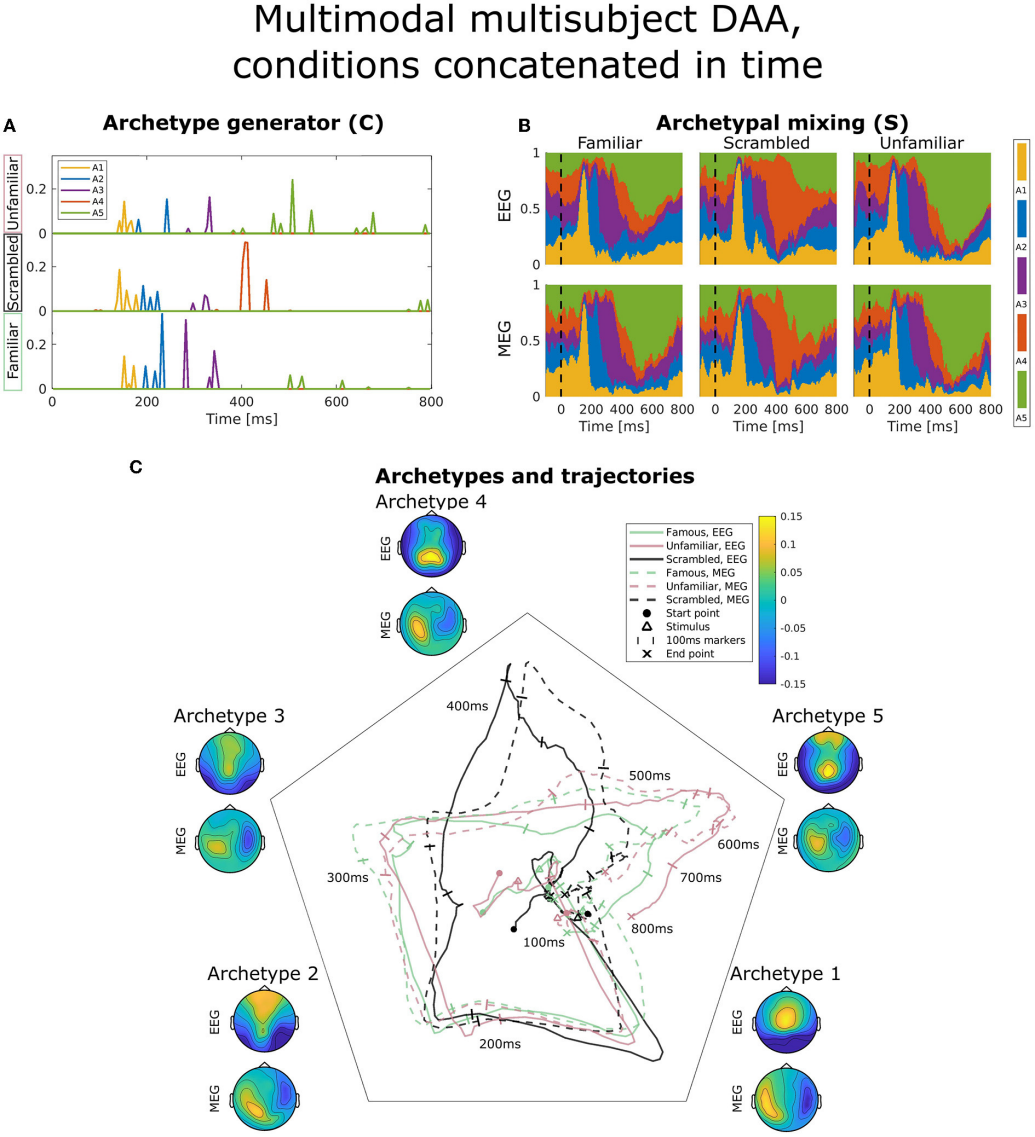
Visualization of the best obtained fit for multimodal, multisubject DAA with *K* = 5 components, where the three conditions have been concatenated in time for all subjects and modalities prior to modeling to enforce equal archetypes. **(A)** The subject- and modality-shared archetype generator matrix **C** with information on the specific samples from which archetypes are generated. The matrix has been split into three parts corresponding to the three conditions and subsequently stacked for visualization purposes. **(B)** The archetypal mixing matrix **S** averaged across subjects showing how samples are probabilistically allocated to archetypes. The mixing matrix has been smoothed with a rectangular window of size 3 samples. **(C)** Archetype trajectory averaged across subjects based on the mixing matrix **S** smoothed with a rectangular window of 10 samples as well as average archetype topographical maps.

**Figure 7:**
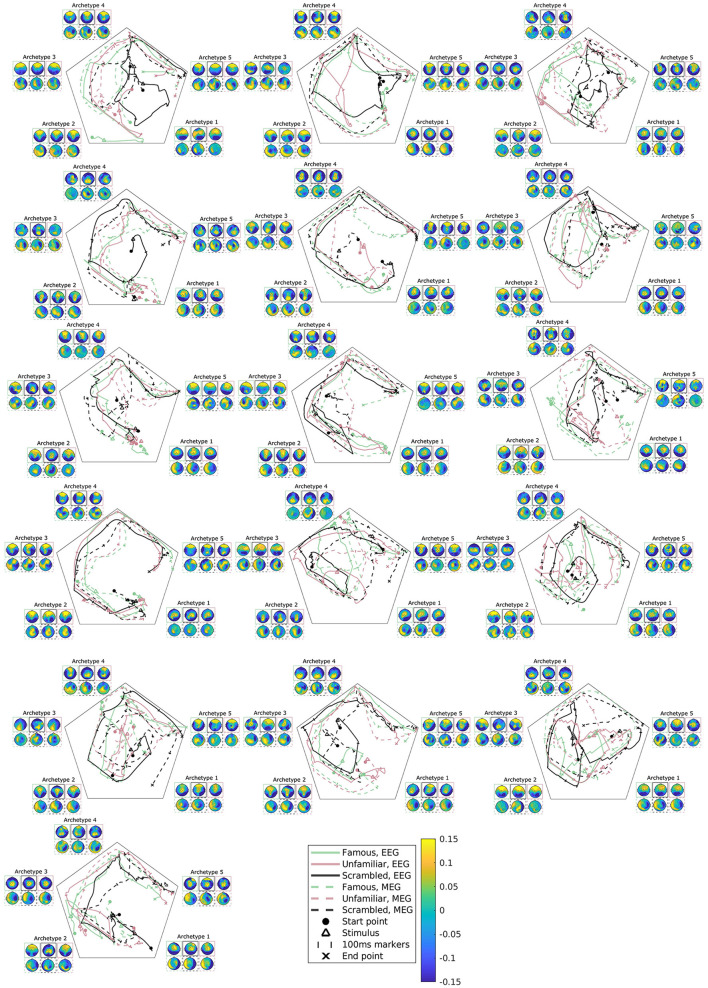
Subject, modality, and condition-specific archetypal trajectories and archetypes for the best multimodal, multisubject DAA model in terms of loss with *K* = 5 components. The matrix **S** has been smoothed with a rectangular window of size 30 samples for visualization purposes. The figures are ordered by subjects, arranged from left to right.

We illustrate the multimodal, multisubject DAA results in Figures 4–7. Once again, we performed runs with an inner loop of 100 initializations to avoid local minima and an outer loop of 5 to estimate run-to-run variability between best-performing solutions. We do not include the Euclidean equivalent as the existing code (Hinrich et al., 2016) does not support fusion of multiple modalities.

Six models were evaluated: (1) a multimodal, multisubject, multicondition DAA where the three conditions (familiar, unfamiliar, scrambled) are modeled similarly to modalities and subject, i.e., with a shared archetype-generating matrix **C** but modality-, subject-, and conditions-specific archetypes ${\tilde{X}}^{\left(m,b,c\right)}C$ and mixing matrices **S**^(*m, b, c*)^, where *c* = {1, 2, 3} is conditions, (2) a multimodal, multisubject DAA where the three conditions were concatenated in time for each subject to enforce equal archetypes $\left[{\tilde{X}}^{\left(m,b,1\right)},{\tilde{X}}^{\left(m,b,2\right)},{\tilde{X}}^{\left(m,b,3\right)}\right]\text{C}$ across conditions but retain separate mixing matrices **S**^(*m, b, c*)^, and (3) a model where there is no correspondence, i.e., each subject, modality, and condition is modeled separately with their own archetype-generating matrix **C**^(*m, b, c*)^. For all three mentioned models, the corresponding model using directional clustering was evaluated.

On Figure 4, we once again observe a steadily decreasing loss with an increasing number of components, and it is difficult to identify a model that constitutes a sound balance between low loss and few components. Generally, we observe lower loss the more flexible the model is. As such, the models where all subjects are modeled separately have a lower loss, while the models where conditions are concatenated in time display the highest loss. Model consistency is generally lower for the zero-correspondence models, and as expected from our synthetic and unimodal analysis, directional clustering consistently performs worse in terms of loss than DAA, with model consistency slightly improved compared to DAA.

In Figure 5A, the archetype generator (**C**) is shown for the best multimodal, multisubject, multicondition DAA model with *K* = 5 archetypes. Since AA constrains **C** to be non-negative, the result is a sparse representation of the post-stimulus time points. As expected, almost none of the archetypes are generated by time points prior to the earliest ERP deflection at about 100 ms. Subsequently, each archetype is generated by a closely located selection of time points, each responsible for a section of the ERP. Interestingly, the late reaction (>600 ms) is covered by a separate archetype, indicating that the late response contains structure beyond the pre-100 ms time points.

Figure 5B shows the archetypal mixing matrix (**S**) averaged across subjects, i.e., the soft assignments of each time-point in the ERP to archetypes. The archetypes have been ordered according to their activation pattern. Archetypal mixing generally follows the pattern in the archetypal generator with little deviation between conditions and modalities. As expected, the prestimulus period until around 100 ms shows no discernible structure. To further investigate archetypal mixing, we show the average ERP trajectory between archetypes in Figure 5C. By arranging the *K* archetypes with equal angle spacing on the unit circle in the plane, we can visualize the trajectory using the mixing coefficients (**S**) for each archetype as coordinates in this plane. Equal expression of all archetypes will be in the middle of the plane, and if one archetype is expressed more than others, the trajectory is dragged toward the corresponding archetype's edge. The trajectory similarly shows fast activation of archetypes 1, 2, and 3 and slower recruitment of archetypes 4 and 5. The path from one archetype to the next involves a general shift in the archetypal activation probability of all other archetypes, i.e., the trajectory curves toward the center of the trajectory space. Generally, we observe indistinguishable trajectories between the two modalities. This was expected since a deviation would indicate that EEG and MEG observed different evoked responses to the same stimuli. For the multimodal, multisubject, and multicondition model, we observe almost no difference in archetypal trajectory between conditions (famous, unfamiliar, scrambled).

Figure 5C also shows the archetype topographical maps averaged across subjects. Upon visual activation, archetype 1 is activated around 100 ms post-stimulus. Archetype 1 is represented by an expected occipital/central dipolarity for EEG corresponding to V1 activation and a strong left/right MEG component. The negative ERP deflection at 170 ms is seen here as a shift from archetype 1 to 2 represented by lateral occipital vs. frontal EEG topography. Wakeman and Henson (2015) commented on a significantly larger ERP component at 170 ms for familiar and unfamiliar faces vs. scrambled faces. Spatially, the authors reported that this difference was significant for frontal electrodes (more positive for familiar and unfamiliar faces compared to scrambled) and lateral occipital electrodes (more negative). Our results show that this difference manifests itself in a stronger lateral occipital vs. frontal activation in EEG topography for familiar and unfamiliar conditions as opposed to scrambled, i.e., very similar results to Wakeman and Henson (2015). This result also falls in line with the general notion that the N170 component corresponds to fusiform gyrus activation for face recognition (Gao et al., 2019). While the corresponding topographic maps for MEG show a larger frontal/occipital polarity, the distinction between conditions is less clear. Archetype 3, which is active at around 300 ms, is a less strong (polarity-wise) version of archetype 2, which does not differ between conditions. Archetype 4 is longer-lasting, dominated by parietal EEG topography. Finally, archetype 5 once again displays strong occipital vs. frontal activation in all three conditions. This corresponds well with the late activation of frontal areas reported in Wakeman and Henson (2015). Generally, we observe similar archetype topographies as observed in the unimodal analysis (Figure 3) with frontal/central/occipital variation in EEG maps and left/right variation in MEG maps.

Figure 6 displays the same visualizations for the model in which conditions have been concatenated in time to enforce equal archetypes between conditions. The result is a **C**-matrix three times the length, which in Figure 6A has been split and stacked to compare condition effects on our model. Interestingly, archetype 4 (red) is purely generated by the scrambled condition (middle), and the late archetype 5 (green) is predominantly generated by the familiar and unfamiliar condition. Archetypal mixing, visible in the mixing matrix visualization on Figure 6B and trajectories on Figure 6C, shows that while the initial part of the ERP displays similar archetypal trajectory between conditions, archetype 4 is almost exclusively visited by the scrambled condition, whereas the familiar and unfamiliar condition visit archetypes 3 and 5. Following fusiform face area activation (archetypes 2 and 3), these results indicate that the familiar and unfamiliar conditions are followed by a late, 400 ms frontal and parietal activation. In contrast, the scrambled condition does not show the frontal component. Smaller differences are present between the familiar and unfamiliar condition seen late, at about 550–600 ms, evident by the unfamiliar condition being dominated by archetype 5 to a greater extent than the familiar condition, especially for the EEG modality.

As previously mentioned, subject variability is high in this data set (Figure 2). The present model accounts for both intermodal and intersubject variability, and accordingly, we can further explore subject variability in the trajectory plots and archetypes of the multimodal, multisubject, multicondition model (see Figure 7). Evidently, subject variability is higher in archetypal mixing than in the archetypes. This makes sense since the archetypes are directly computed from the same convex combinations of the input data. As such, the variability in input data is propagated to the archetypes.

Most of the subjects follow an archetypal trajectory pattern that starts centrally and, approximately 100 ms after stimulus, travels to archetype 1, and then quickly onto archetype 2-5 in a circular pattern. However, some conditions for some subjects fall outside this pattern (see, once again, subjects 3, 6, and 9). The scrambled condition does not appear to be the cause of these deviations. Likewise, there is little visible difference between trajectories for EEG and MEG.

## 4. Discussion

We have presented the directional archetypal analysis (DAA) for scale- and polarity-invariant modeling of brain microstates and demonstrated its utility in modeling both unimodal and multimodal M/EEG ERP data from a visual perception task. We validated our models on synthetic data, compared results to the conventional Euclidean AA model, and showed that DAA, unlike Euclidean AA, can efficiently characterize antipodally symmetric, spherical data. Our unimodal analyses showed that DAA loss as a function of the number of components (*K*) saturated earlier when compared to its Euclidean counterpart, although loss functions are not directly comparable. Notably, Euclidean AA potentially computes archetypes corresponding to the dipole counterpart of other archetypes. We further observed that the Euclidean AA was more affected by the scale of the input data. The scale of the learned archetypes were inherently equal for DAA but the Euclidean AA produces archetypes with highly varying scales. However, if the underlying convex hull generating the data are obscured by the projection onto the sphere, i.e., by including the origin as an archetype, the directional models cannot viably model the data.

We contrasted DAA to a clustering model that constrain the archetypal mixing matrix **S** to hard assignment, corresponding to a multimodal and multisubject extension of the modified *k*-means procedure (Pascual-Marqui et al., 1995). Our synthetic example showed that a clustering model might be suitable for polarity invariant data, although it determines prototypes, or centroids, defining *typical* points. In contrast, DAA identifies archetypes constituting representative, extremal points of the data set. This clustering model is akin to conventional brain microstate analyses, which employ a polarity-invariant *k*-means approach allowing component correspondence while accounting for spatiotemporal variability. While such a model is useful, it also heavily simplifies the notion of brain states to be a one-at-a-time phenomenon. This approach has recently been challenged by Mishra et al. (2020) who suggested that the brain traverses microstates in a continuous rather than discrete pattern. Our proposed DAA approach is a potential solution to this problem by determining microstates based on archetypes rather than prototypes and estimating a (continuous) mixing matrix based on the archetypes. With the added flexibility, we also observed that our model leads to improved loss compared to the corresponding clustering formulation highlighting how the model representation provides more detailed characterizations of the data.

DAA is readily extended to both multisubject and multimodal modeling. Here, we approached the problem by estimating a shared archetype generator matrix **C** and subject and modality-specific archetype mixing matrix **S**^(*m, b*)^. Importantly, the archetypes ${\tilde{X}}^{\left(m,b\right)}C$ themselves are subject and modality-specific since they are constructed through convex combinations of the input data. In our analyses of ERP data from several conditions (familiar, scrambled, unfamiliar), we extended this approach to also account for conditions. As such, each condition was treated as a new subject to get subject, modality, and condition-specific archetypes and mixing matrices. Our model (Figure 5) showed some variation between conditions observed in an archetype active at approximately 200 ms with stronger bilateral occipital vs. frontal polarity for familiar and unfamiliar compared to scrambled faces. These results were in line with a previous study on the same data set (Wakeman and Henson, 2015) and are also consistent with the general N170 ERP peak representing fusiform gyrus activation specific for face recognition (Gao et al., 2019). Another solution to having multiple conditions is to concatenate these over time and thus allow the archetype generator (**C**) to be driven by specific condition(s) and not necessarily the same time points across conditions. This approach showed a clear distinction between scrambled and the two face conditions. Specifically, one of the five archetypes was purely generated and visited by the scrambled condition, while two others were mostly generated and visited by the familiar and unfamiliar conditions. Larger frontal activation in face conditions has been observed previously on the same data set (Wakeman and Henson, 2015; Quinn et al., 2018).

By having a shared archetype-generator matrix **C** across subjects, modalities, and conditions, we implicitly assume that the *timing* of the neural response to stimuli is the same. This assumption is valid across modalities since these were acquired simultaneously and thus measured the same underlying response. Similarly, it would be expected that the timing is similar for multiple stimuli for the same subject; however, the assumption of zero latency might not be valid across subjects. One solution to this problem is to employ an even more flexible model that does not assume any correspondence between subjects, modalities, or conditions. As highlighted in Figure 4, such a model would lead to improved loss. However, it is much more difficult to establish component correspondence and infer population-level archetypes and archetypal trajectory behavior. We expect that future work may look into developing latency-invariant models inspired by shift-invariant decompositions (Mørup et al., 2008). The zero-latency assumption currently limits the extension of our framework to continuous data, such as resting-state. Similarly, multimodal fusion with vastly different modalities, such as fMRI, which usually measures slow blood-oxygen response to stimuli, and EEG or MEG would violate the assumption of equal timing of the neural response.

A multisubject AA framework, first presented in Hinrich et al. (2016), allows us to account for subject variability, which we know is present in the data set under consideration (see Figure 2). Figure 7 displays the estimated subject-specific archetypes and trajectories and shows that generally, the subject variability manifests itself in archetypal trajectories. Archetype topographies generally also vary across subjects, however, not to the same degree. This highlights the importance of accounting for spatiotemporal variability.

In our analyses on real data, we did not observe a corner point, or clear bend, in the loss curves that would otherwise indicate a potential optimal number of archetypes for any of the evaluated models. Future work may consider cross-validation for model selection. Specifically, we believe that a split-half setup, in which trials are randomly split into two groups prior to preprocessing, where one group is used for training the model and the other for evaluating model loss, is favorable. When the number of trials is high, split-half ensures all subjects and conditions are represented in both groups and high SNR in the corresponding averages while avoiding the excessive computational demands of, e.g., K-fold cross-validation.

Archetypal analysis is generally prone to local minima, a characteristic we also observed in our analyses. All presented loss curves were averages of 5 runs, each the best of 100 different initializations. This higher number of initializations also affected the presented Euclidean AA results (i.e., Figures 1, 3). Generally, we observed that model consistency for DAA was slightly improved by increasing the number of models in the inner loop from 20 to 100, while for conventional AA, model consistency was generally lower than DAA for 20 models in the inner loop and very high for 100 models. While we did not present these results, we argue that all AA models, whether directional or not, benefit from evaluating multiple initializations. For 100 runs in the inner loop, especially for the synthetic data set, Euclidean AA showed higher NMI than DAA, which shows that the robustness of DAA may be somewhat challenged. While it has been shown that the optimization of **C** and **S** individually is convex for a least-squares loss function, this property breaks down for the proposed Watson equivalent due to the normalization term projecting the reconstruction to the sphere. As a result, we hypothesize that the reduced DAA robustness compared to least-squares for 100 runs could be a consequence of the optimization landscape being more prone to local minima issues.

Here we initialized our models by random sampling from an exponential distribution. Previous studies have shown that initializing **C** as carefully selected samples through the *FurthestFirst* (Cutler and Breiman, 1994) or the improved *FurthestSum* (Mørup and Hansen, 2012), may lead to improved convergence speed. However, over multiple initializations, random initialization has been shown to lead to lower losses (Krohne et al., 2019). Further studies could evaluate the effect of initialization to potentially decrease the number of estimated models needed to ensure robustness of the obtained results.

Directional archetypal analysis and clustering assume that data resides on a (unit) hypersphere. In our case, the dimensionality of the hypersphere corresponds to the number of electrodes and magnetometers, respectively. AA, including DAA, allows for the archetypes $\tilde{X}\text{C}$ to be constructed from a data matrix $\tilde{X}$ potentially different from the original data matrix **X**. Here, we constrained each sample of the input data ${\tilde{X}}^{\left(m,b\right)}$ to unit *l*_2_-norm, while **X** was normalized by the Frobenius norm of all samples across all subjects, conditions, and modalities, to ensure that these were given similar influence on the model. Normalization of $\tilde{X}$ by the *l*_2_-norm was our approach to scale and polarity invariant modeling of microstates. Optimization of **C** and **S** occurred with a loss function of the reconstruction (using ${\tilde{x}}_{n}$) to the original, unnormalized data **x**_*n*_. In this way, the squared magnitude of the data, interpreted as the precision parameter κ absorbed by **x**_*n*_, enabled DAA to emphasize regions with high SNR when defining the archetypes. This is similar to how conventional microstate analysis procedures typically restrict the analysis to regions of high global field power (Poulsen et al., 2018).

In conclusion, we have introduced directional archetypal analysis for (1) modeling of scale and polarity invariant data, (2) fusion of multiple modalities, and (3) incorporating subject variability in archetypes and archetypal mixing. Our model represents an approach to modeling brain microstates without assuming hard assignment of states to samples that accounts for spatiotemporal variability of the brain's response to stimuli while preserving component correspondence.

## Data availability statement

Publicly available datasets were analyzed in this study. The code for DAA as well as the hard assignment multimodal multisubject clustering procedure and further information regarding the experiments are available at https://github.com/anders-s-olsen/DirectionalArchetypalAnalysis. The data may be freely downloaded at https://openneuro.org/datasets/ds000117/versions/1.0.4.

## Ethics statement

The studies involving human participants were reviewed and approved by Cambridge University Psychological Ethics Committee. The patients/participants provided their written informed consent to participate in this study.

## Author contributions

AO and RH: methodology, software, validation, formal analysis, data curation, visualization, writing—original draft, and writing—review and editing JH: conceptualization, methodology, supervision, and writing—review and editing. KM: methodology, supervision, and writing—review and editing. MM: conceptualization, formal analysis, methodology, project administration, software, supervision, writing—original draft, and writing—review and editing. All authors contributed to the article and approved the submitted version.

## Funding

Through RH, this work was partly funded by the Innovation Fund Denmark (IFD, grant number: 9065-00077B). MM was supported by Ingeborg and Leo Dannins scholarship for scientific research.

## Conflict of interest

Author RH is employed by WS Audiology. The remaining authors declare that the research was conducted in the absence of any commercial or financial relationships that could be construed as a potential conflict of interest.

## Publisher's note

All claims expressed in this article are solely those of the authors and do not necessarily represent those of their affiliated organizations, or those of the publisher, the editors and the reviewers. Any product that may be evaluated in this article, or claim that may be made by its manufacturer, is not guaranteed or endorsed by the publisher.

## Footnotes

^1^ https://openneuro.org/datasets/ds000117/versions/1.0.4

^2^ https://github.com/robertoostenveld/Wakeman-and-Henson-2015

^3^See e.g., https://tminka.github.io/papers/matrix/minka-matrix.pdf.
